# The Use of Mixed Populations of *Saccharomyces cerevisiae* and *S. kudriavzevii* to Reduce Ethanol Content in Wine: Limited Aeration, Inoculum Proportions, and Sequential Inoculation

**DOI:** 10.3389/fmicb.2017.02087

**Published:** 2017-10-25

**Authors:** Javier Alonso-del-Real, Alba Contreras-Ruiz, Gabriel L. Castiglioni, Eladio Barrio, Amparo Querol

**Affiliations:** ^1^Departamento de Biotecnología de los Alimentos, Grupo de Biología de Sistemas en Levaduras de Interés Biotecnológico, Instituto de Agroquímica y Tecnología de los Alimentos (CSIC), Valencia, Spain; ^2^Departament de Genètica, Universitat de València, Valencia, Spain

**Keywords:** *Saccharomyces* yeast, wine fermentation, ethanol reduction, fermentation oxygenation, starter cultures

## Abstract

*Saccharomyces cerevisiae* is the most widespread microorganism responsible for wine alcoholic fermentation. Nevertheless, the wine industry is currently facing new challenges, some of them associate with climate change, which have a negative effect on ethanol content and wine quality. Numerous and varied strategies have been carried out to overcome these concerns. From a biotechnological point of view, the use of alternative non-*Saccharomyces* yeasts, yielding lower ethanol concentrations and sometimes giving rise to new and interesting aroma, is one of the trendiest approaches. However, *S. cerevisiae* usually outcompetes other *Saccharomyces* species due to its better adaptation to the fermentative environment. For this reason, we studied for the first time the use of a *Saccharomyces kudriavzevii* strain, CR85, for co-inoculations at increasing proportions and sequential inoculations, as well as the effect of aeration, to improve its fermentation performance in order to obtain wines with an ethanol yield reduction. An enhanced competitive performance of *S. kudriavzevii* CR85 was observed when it represented 90% of the cells present in the inoculum. Furthermore, airflow supply of 20 VVH to the fermentation synergistically improved CR85 endurance and, interestingly, a significant ethanol concentration reduction was achieved.

## Introduction

Wine composition is the product of complex interactions among yeast and bacteria that take place in vineyards and wineries, although one yeast species, *Saccharomyces cerevisiae*, is generally the main microorganism responsible for winemaking process (Pretorius, 2000). Its vigorous fermentative capacity, even in the presence of oxygen (Crabtree effect), makes *S. cerevisiae* a very efficient ethanol producer, strategy that allows its imposition over the rest of the microbiota during fermentation due to the toxicity of this compound (Thomson et al., 2005; Piškur et al., 2006).

However, this high ethanol production capability may be disadvantageous taking into account the challenges currently faced by the wine industry. In the first place, global warming provokes a gap during grape ripening between phenolic maturity and sugar content. If grapes are harvested when the sugar content is appropriate but the phenolic maturity has not been reached, wines can show altered aroma, flavor, mouth feel, and astringency. On the contrary, if grapes are harvested when their phenolic maturity is the appropriate, their sugar contents are higher, giving rise to wines with increasing ethanol concentrations (Jones et al., 2005). This higher ethanol content is undesirable according to consumers' new demands, because affects flavor complexity sensing (Goldner et al., 2009), and its excessive consumption is harmful for health and road safety.

A variety of measures are taken at the different winemaking stages to overcome the problem of the higher ethanol levels in wines. These include new agronomical methods for grape cultivation (Intrigliolo and Castel, 2009), the use of mixed musts from grapes at different ripening stages (Kontoudakis et al., 2011), the use of engineered yeasts producing lower ethanol yields (Varela et al., 2012), or the partial dealcoholisation of wines by chemical or physical procedures (Gómez-Plaza et al., 1999; Pilipovik and Riverol, 2005; Diban et al., 2008; Hernández et al., 2010; Offeman et al., 2010; Belisario-Sánchez et al., 2012). However, some of these approaches have little impact on ethanol contents, negatively affect the quality of wine, are highly expensive industrial processes, or contravene the current regulations about the use of GMO.

In addition, a wide range of different biological strategies have been proposed to reduce alcohol contents in wines (Kutyna et al., 2010). The use of non-conventional yeast strains in winemaking stands out for its potential. Several non-*Saccharomyces* yeasts, usually in combination with *S. cerevisiae*, have been tested to reduce ethanol yields during wine fermentation (Comitini et al., 2011; Sadoudi et al., 2012; Contreras et al., 2014, 2015; Quirós et al., 2014; Ciani et al., 2016). Different strategies have been carried out to improve the fermentation performance of these non-*Saccharomyces* yeasts, such as, sequential inoculation or co-inoculation at increased proportions with *S. cerevisiae*, to provide new characteristics to the final wines (Andorrà et al., 2012; Gobbi et al., 2013; Izquierdo Cañas et al., 2014; Jolly et al., 2014; Loira et al., 2014; Canonico et al., 2016). Another approach to reduce alcohol content in wines is the supply of oxygen to the fermenters, under a controlled flowrate, to promote the respiratory consumption of sugars by these non-*Saccharomyces* yeasts (Gonzalez et al., 2013; Rodrigues et al., 2016). However, temperature under industrial winemaking conditions is generally close to 25°C, which does not allow for any of these alternative yeasts to survive the first hours of the process (Nissen and Arneborg, 2003; Torija, 2003; Pérez-Nevado et al., 2006; Williams et al., 2015).

Alternative *Saccharomyces* yeasts, such as, *Saccharomyces kudriavzevii* or *S. uvarum*, can help to solve some of the new challenges of the wine industry. These species exhibit physiological properties that are especially relevant during the winemaking process, such as, their good fermentative capabilities at low temperatures, resulting in wines with lower alcohol and higher glycerol amounts (Varela et al., 2016; Pérez-Torrado et al., 2017a). In the case of *S. kudriavzevii*, this species displays a different metabolic regulation concerning ethanol and glycerol syntheses (Arroyo-López et al., 2010; Oliveira et al., 2014; Pérez-Torrado et al., 2016). Moreover, it recently showed an ethanol reducing capability in mixed fermentation with *S. cerevisiae* at low temperatures (Alonso-del-Real et al., 2017). Again, temperature appears as the most important factor to determine the preponderance of *S. cerevisiae* during wine fermentation (Nissen and Arneborg, 2003; Torija, 2003; Pérez-Nevado et al., 2006; Arroyo-López et al., 2011; Salvado et al., 2011; Williams et al., 2015; Alonso-del-Real et al., 2017).

However, none of the techniques used to favor the growth of non-*Saccharomyces* yeasts, such as, co-inoculation, sequential inoculation, or microoxigenation, have been applied to *S. kudriavzevii* species to favor their presence during wine fermentation. In this work, we first analyzed the presence of *S. kudriavzevii* during co-fermentation with a *S. cerevisiae* wine strain under different aeration conditions to select the most suitable one. Next, we studied the effect of *S. kudriavzevii* enrichment in the inoculum with and without external oxygen supply, and finally the effect of sequential inoculation of the strains.

## Materials and methods

### Yeast and growth media

The commercial *S. cerevisiae* strain T73 (Lalvin T73 from Lallemand Monteral, Canada), was used as a conventional wine strain. *S. kudriavzevii* CR85, a natural isolate from oak tree bark in Agudo, Ciudad Real province, Spain, was selected as the non-conventional, quality enhancer candidate yeast according to its physiological properties. In a recent study, CR85 was shown to be the *S. kudriavzevii* strain with better fermentation kinetics, despite the high genomic homogeneity among that species (Peris et al., 2016).

Synthetic must (SM, Rossignol et al., 2003) was used in microvinification experiments, with 100 g/L glucose and 100 g/L fructose. YPD medium (2% glucose, 2% peptone, 1% yeast extract) was used for overnight growth of precultures.

### Synthetic must fermentations

First, in order to determine the best aeration condition, fermentations of 200 mL SM were carried out by a *S. cerevisiae* and *S. kudriavzevii* co-inoculum (ratio 1:1) at four different aeration conditions throughout the process: 1 VVH, 5 VVH, 10 VVH, and 20 VVH taking in account the previous data from non-conventional yeasts (Morales et al., 2015). Secondly, different ratios *S. cerevisiae*/*S. kudriavzevii* (1:1, 3:7, and 1:9) were used in further 200 mL SM fermentations, both in anaerobiosis and with an air flow rate of 20 VVH during the first 48 h. Also, a condition in which *S. cerevisiae* was inoculated after 24 h in a proportion of 1% with respect to *S. kudriavzevii* was also considered. Single cultures of *S. cerevisiae* and *S. kudriavzevii* were taken as control for fermentation. In addition, a bottle containing distilled water and another one with water and 5% (v/v) ethanol were set as control for evaporation and ethanol loss due to aeration.

Aeration system is composed of a compressed air generator, 3.1 mm internal diameter silicon tubes, 0.2 μm pore-size filters, a flow meter and a set of flow regulators (one for each bottle) as depicted in Supplementary Figure 1. All the experiments were conducted in triplicate at 25°C with gentle shaking (100 rpm) and an initial inoculation with an OD_600_ of 0.2. The fermentation process was monitored through weight loss. Yeast cells were collected at different moments during fermentation and kept at −20°C to determine the proportion of both yeast species by QPCR, according to Alonso-del-Real et al. (2017). Supernatants of the samples were also stored at −20°C for the analysis of wine composition by HPLC.

### HPLC analysis and data treatment

Sugars (glucose and fructose), glycerol, ethanol, and acetic acid from the fermentation at different time point samples were determined by HPLC (Thermo Fisher Scientific, Waltham, MA, USA) using a refraction index detector and a HyperREZTM XP Carbohydrate H + 8 μm column (Thermo Fisher Scientific) equipped with a HyperREZTM XP Carbohydrate Guard (Thermo Fisher Scientific). Samples were 3-fold diluted, filtered through a 0.22-μm nylon filter (Symta, Madrid, Spain) and injected in duplicate. The analysis conditions were: eluent, 1.5 mM of H_2_SO_4_; 0.6 ml min-1 flux and a 50°C oven temperature.

Water and ethanol losses were considered as lineal with respect to time. Deviation factors were dimensioned in bottles with 5% (w/v) ethanol in 400 mL water, and bottles with 400 mL of water, all them with air supply (20 VVH). Water mass loss followed a lineal equation (*R*^2^ = 0.99569):

(1) y = 0.1684t

where *y* refers to weight loss due to H_2_O evaporation in bottles with only water and *t* refers to time.

(2) y = 0.2532t

where *y* refers to weight loss due to H_2_O and ethanol evaporation in bottles with 5% (w/v) ethanol and *t* refers to time. HPLC measures of the last were taken at different time points. We observed that ethanol loss followed a lineal function, and that a subtraction of the equation for ethanol bottle minus the one for water bottle, very precisely predicted HPLC results. The calculation was done following Equations (3–5):

(3) $$F_{1}=\frac{\left((a_{1}-a_{2})\times 100\right)}{20}$$

where *F*_1_ is factor 1 for ethanol correction (% h^−1^), *a*_1_ is the slope of Equation (1), *a*_2_ is the slope of Equation (2), and 20 is the value for the total mass of ethanol weighted for 400 mL of solution.

(4) $$F_{2}=\frac{(F_{1}\times t)\times E_{HPLC}}{20}$$

where *F*_2_ is factor 2 for ethanol correction (%), *t* is the time corresponding to an assessed value and *E*_*HPLC*_ is the HPLC measure for ethanol concentration.

(5) $$E_{C}=\frac{\left(F\_2+E\_HPLC\;).[V\_T-(a2\times t)\right]}{V_{T}}$$

where *E*_*C*_ is corrected ethanol concentration (%).

The rest of compounds in our system were assumed as nonvolatile, however, their concentration values were considered as affected by water and ethanol volume losses. To calculate this concentration factor, the density of must was considered to be equal to the density of water. HPLC values for glucose, fructose, glycerol and acetic acid were corrected using the following equation:

(6) $$C_{C}=\frac{C_{HPLC}\times 1000}{(1000+\left(a_{2}\times t\right)+\left[(E_{C}-E_{HPLC})\times 10\right]}$$

where *C*_*C*_ is the corrected concentration for the compound.

Fermentations were tested for the significant differences among them with an ANOVA using the one-way ANOVA module of the Statistica 7.0 software. The concentrations of glucose, fructose, glycerol, ethanol, and acetic acid obtained by HPLC were introduced as the dependent variables. Means were grouped using the Tukey HSD test (α = 0.05).

## Results

### Determining the air flow conditions favoring *S. kudriavzevii* presence in mixed fermentations with *S. cerevisiae*

**Figure 1:**
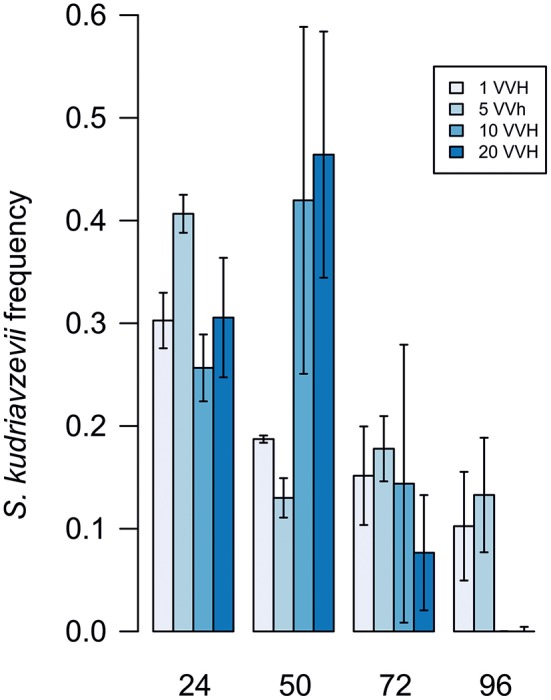
*S. kudriavzevii* frequency under different aeration conditions. Values are mean of three replicates. Error lines represent standard deviations.

A controlled aeration system feeding a set of fermentations co-inoculated with *S. cerevisiae* and *S. kudriavzevii* in a ratio 1:1 with 4 different air flow rates: 1, 5, 10, and 20 VVH was installed. Figure 1 shows a clear disadvantage of *S. kudriavzevii* even in the presence of an external oxygen input. However air flow rate seems to have an influence on the time that *S. kudriavzevii* can remain in the culture in substantial proportions, and thus, can have a more relevant role during fermentation. The percentage of *S. kudriavzevii* was higher than 30% during the first 48 h in fermentations performed with air flows of 10 and 20 VVH. However, after 48 h of fermentation a faster decline of the *S. kudriavzevii* population is observed, which suggests that aeration only favors *S. kudriavzevii* growth at the beginning of the fermentations.

### Assaying different *S. cerevisiae/S. kudriavzevii* inoculation proportions in fermentations with and without air supply

According to these previous data, aeration was applied only for short periods (48 h) for subsequent fermentations because longer aeration time does not favor growth of *S. kudriavzevii*, and also could increase the final acetic acid concentrations in wines, due to respiration (Salmon, 2006). To test whether a higher inoculation from the beginning of the fermentation, in combination with aeration, could improve *S. kudriavzevii*'s competitive performance, starters composed by *S. cerevisiae*/*S. kudriavzevii* proportions of 1:3 and 1:9 in were inoculated into fermentations supplied with an air flow rate of 20 VVH during the first 48 h. Fermentations in the same conditions without aeration were also included to analyze the effect of the yeast species proportions alone.

There were significant differences between aerated and non-aerated fermentations. First, there is a considerable reduction of the fermentation time at which all sugars were totally consumed. Whereas unaerated fermentations took 10 days to finish, aerated fermentations took only 7 days. Second, a clear effect on the maximum cell density was observed, thus, single cultures of *S. cerevisiae* and *S. kudriavzevii* with air supply reached OD_600_ values around 25, however OD_600_ values for single cultures without aeration were around 20 and 15, respectively.

**Figure 2:**
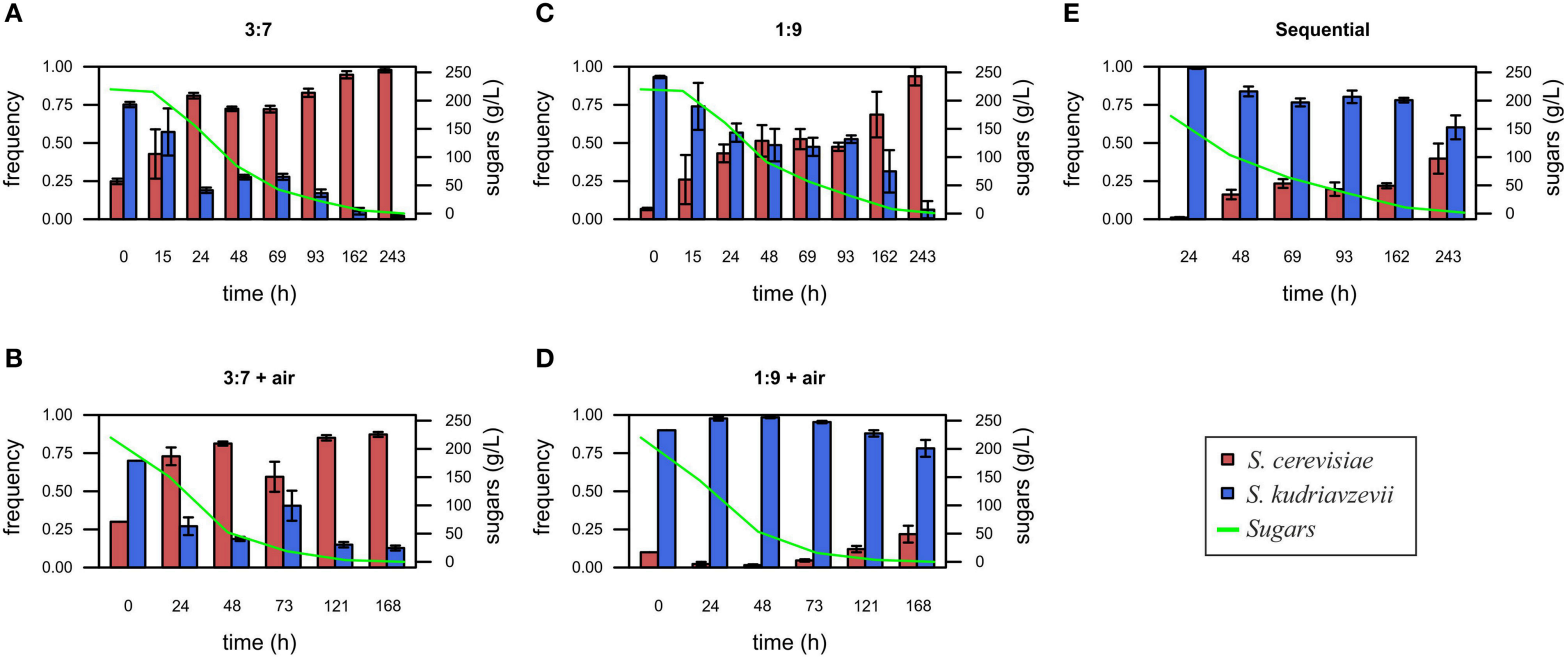
*Saccharomyces cerevisiae* and *S. kudriavzevii* frequency during fermentation under different conditions: inoculum proportion 3:7 without air **(A)**, inoculum proportion 3:7 with aeration during the first 48 h **(B)**, inoculum proportion 1:9 without air **(C)**, inoculum proportion 1:9 with aeration during the first 48 h **(D)**, and sequential inoculation **(E)**. Values are mean for 3 replicates. Error bars represent standard deviations. The sum of glucose and fructose concentrations in the must at every time point was also shown.

Regarding yeast proportion changes during fermentations, the initial inoculum proportion of 1:3 shows a slight increase of the frequency of *S. kudriavzevii* at the final fermentation stage due to limited air supply (Figures 2A,**B**). However, this inoculation ratio does not provide, with respect to the 1:1 proportion a clear competition advantage for *S. kudriavzevii*. However, when the inoculation proportion was 1:9 and without aeration (Figure 2C), *S. kudriavzevii* is able to remain at frequencies higher than 40% for 4 days, although at the end, is outcompeted by *S. cerevisiae*. Strikingly, the addition of the oxygen supply to inoculation proportions of 1:9 seems to provide a favorable environment for *S. kudriavzevii* imposition (Figure 2D).

Sequential inoculation is one of the most common strategies proposed for the preservation of non-dominant microorganisms during food fermentations (Gobbi et al., 2013; Contreras et al., 2014; Loira et al., 2014). In the present study, this strategy was also applied by inoculating a set of bottles only with *S. kudriavzevii* at the beginning, and adding *S. cerevisiae* after 24 h in a proportion of 1%. In this case, *S. cerevisiae* was able to increase its frequency to 40% at the end of the fermentations (Figure 2E).

As a summary of these results, the use of aeration has a slight impact on the relative competitive fitness of *S. kudriavzevii* when inoculated at equal proportions with *S. cerevisiae*. However, highly biased proportions of *S. kudriavzevii*, as well as sequential inoculations, can extend the presence of this less competitive species of interest to promote its impact in the fermentation process. Nevertheless, the combination of aeration and biased inoculation synergistically improves *S. kudriavzevii* presence during fermentation.

### Effect of the different inoculation-aeration strategies on the final fermentation product

To determine if these strategies really improve wine fermentations, the final wine composition was evaluated by HPLC analysis. First, it is important to remark that in all assayed conditions fermentations were finished with the consumption of all sugars present in the original must, except for fermentations performed only with single cultures of *S. kudriavzevii* (Table 1), and under aeration, fructose was totally consumed.

**Table 1:** Chemical composition of the fermented SM obtained through HPLC.

***Sce*: *Sku* proportion**	**Aeration (VVH)**	**Glucose (g/L)**	**Fructose (g/L)**	**Glycerol (g/L)**	**Ethanol (%)**	**Acetic acid (g/L)**
1:0	0	0.00 ± 0.00^a^	0.12 ± 0.03^a^	5.86 ± 0.11^a, b^	13.13 ± 0.09^a, c^	1.05 ± 0.01^a^
0:1	0	0.02 ± 0.03^a^	4.11 ± 2.34^b^	7.73 ± 0.46^d^	12.50 ± 0.26^a, b^	1.27 ± 0.03^a^
1:1	0	0.00 ± 0.00^a^	0.00 ± 0.00^a^	6.24 ± 0.29^a^	13.27 ± 0.50^c^	1.16 ± 0.13^a^
3:7	0	0.00 ± 0.00^a^	0.15 ± 0.10^a^	6.13 ± 0.09^a, b^	13.04 ± 0.05^a, c^	1.15 ± 0.03^a^
1:9	0	0.00 ± 0.00^a^	0.78 ± 0.75^a^	6.53 ± 0.12^a^	13.00 ± 0.16^a, c^	1.22 ± 0.01^a^
Sequential	0	0.00 ± 0.00^a^	1.63 ± 0.18^a^	7.47 ± 0.21^c, d^	12.46 ± 0.08^a, b^	1.13 ± 0.05^a^
1:1	20	0.00 ± 0.00^a^	0.00 ± 0.00^a^	5.36 ± 0.40^b^	12.12 ± 0.33^b^	1.57 ± 0.10^b^
3:7	20	0.00 ± 0.00^a^	0.00 ± 0.00^a^	6.24 ± 0.55^a^	12.09 ± 0.18^b^	1.61 ± 0.23^b^
1:9	20	0.00 ± 0.00^a^	0.00 ± 0.00^a^	6.61 ± 0.07^a, c^	11.26 ± 0.19^d^	1.79 ± 0.02^b^

*Values are given as mean ± standard deviation of three biological replicates and two HPLC detection runs. An ANOVA analysis was carried out. The values followed by different superindexes in the same column are significantly different according to the Tukey HSD test (α = 0.05)*.

Glycerol concentrations were clearly higher in all conditions in which *S. kudriavzevii* is present, compared to fermentations performed only with the reference *S. cerevisiae* wine strain, except for the 1:1 proportion with aeration. This glycerol production increase was especially relevant in fermentations with sequential inoculation (Table 1).

Ethanol reduction was accomplished in fermentations with microaeration (up to 1.9% v/v less) and with sequential inoculation (Table 1). However, the ethanol reduction achieved by increasing respiration rate had the counterpart of an acetic acid content increase between 0.5 and 0.7 g/L in bottles under limited aeration, which was not observed in non-aerated fermentations.

## Discussion

In the last century, alcohol abuse became considered as one of the most important health problems in the world, and promoted new behavioral strategies against alcohol consumption. In addition, because of global warming, in wine-growing regions with a Mediterranean climate there is excessive ripening of the grape, which produces musts with a higher concentration of sugars (Jones et al., 2005), and hence, higher alcohol yields, implying a higher tax burden, which makes wines less competitive, and a rejection by the consumer for health reasons, road safety, etc.

Therefore, wine industry must respond to these challenges posed both by new consumer demands and by changes in the composition and properties of the grape must due to climate change. These demands have a significant impact on the quality and acceptance of the final wines and require improvements in the enological practices, among which the development of new yeast starters exhibiting lower ethanol yields during wine fermentation is of chief importance.

Different approaches in the use of yeast starters have been proposed to reduce alcohol contents in wines (Schmidtke et al., 2012; Varela et al., 2015). They include controlled aeration, starter strain proportion adjustment, or inoculation of dominant yeast species after a non-*Saccharomyces* yeast of interest (Comitini et al., 2011; Sadoudi et al., 2012; Contreras et al., 2014, 2015; Quirós et al., 2014; Ciani et al., 2016). In the present study, we adapted these strategies to foster a *Saccharomyces* non-*cerevisiae* strain (*S. kudriavzevii* CR85) presence in synthetic must fermentation. This yeast had been proved to foster decreased ethanol content, and also to increase fermentation kinetics and glycerol concentration in a 1:1 inoculum proportion with *S. cerevisiae* under low temperatures conditions. In contrast, this effect was not found under regular red winemaking temperatures (Alonso-del-Real et al., 2017), probably due to some of the already proposed competition mechanisms, such as, antimicrobial GAPDH-derived peptides produced by *S. cerevisiae* (Branco et al., 2016), lower sulfite tolerance and efflux capacity (Pérez-Torrado et al., 2017b), or early nutrient depletion by *S. cerevisiae* (Fleet, 2003). However, the results reported in the present work show that *S. kudriavzevii* presence during an important period of the fermentation was achieved at regular industrial temperatures.

Although *S. kudriavzevii* and *S. cerevisiae* show long-term Crabtree effect, the carbon flux ratio between respiration and fermentation under aerobic conditions seem to be slightly higher in *S. kudriavzevii* CR85 compared to *S. cerevisiae* T73 (our unpublished data). Thus, an external oxygen supply to a fermentation co-inoculated with these two yeast species may benefit *S. kudriavzevii* growth. Nevertheless, high oxygen levels can deteriorate important compounds of must, originating undesired metabolites correlated to respiration such as acetic acid (Salmon, 2006). Therefore, a fine tuning of the amount of oxygen introduced into the system seems to be critical for the final wine quality. A wide range of airflow rates, from 2.4 to 60 VVH have been used at laboratory scale (Vilanova et al., 2007; Shekhawat et al., 2016). Nevertheless, an air flow rate of 20 VVH has been showed to be on the top limit for acetic acid production when applied to *S. cerevisiae* microvinification (Morales et al., 2015), therefore the screening for the most suitable condition was performed always below this value.

*S. kudriavzevii* performance under air supply conditions was observed to improve its competitive fitness against *S. cerevisiae* (Arroyo-López et al., 2011; Alonso-del-Real et al., 2017). Our results suggest, though, that despite maintaining an air supply during the whole fermentation, after 48 h, *S. kudriavzevii* was outcompeted by *S. cerevisiae*. This, together with the fact that an aerobic environment produces a higher acetic acid accumulation up to 70%, led us to reduce aeration just for the first 48 h of fermentation for the successive experiments. Nevertheless, it is noteworthy that, as observed by Moruno et al. (1993) and later confirmed by Beltrán et al. (2008), synthetic and natural musts have different impact on the final product composition, acetic acid levels are much higher for synthetic must, as can also be observed for our aerated conditions. Thus, due to laboratory experimental conditions, acetic acid values obtained in the present work are high even for non-aerated synthetic must fermentations performed with the *S. cerevisiae* wine strain, compared to natural must fermentation under industrial conditions (0.35 g/L). Therefore, acetic acid levels produced during fermentations with air supply could still be under the limits of regulation (~1 g/L) and consumers' acceptance when tested at industrial scale.

Despite the acetic acid increase, ethanol reduction is notable for the aerated fermentations, in concordance with previous studies (Morales et al., 2015; Shekhawat et al., 2016), and similar to ethanol reductions obtained in other works in which similar co-inoculation strategies with non-*Saccharomyces* yeasts have been followed (Contreras et al., 2015; Ciani et al., 2016; Englezos et al., 2016). However, this is the first study in which *S. kudriavzevii* was used to reduce ethanol yields, which, together with a recent study on the sequential inoculation of *S. uvarum* and *S. cerevisiae* (Varela et al., 2016), opens new approaches to the use of other *Saccharomyces* species. These species, in addition to their ethanol metabolic characteristics, also provide richer aroma profiles to wine (Stribny et al., 2015).

The analysis of the non-aerated fermentations also showed a slight ethanol yield reductions clearly correlated with the *S. kudriavzevii* proportions during the fermentation process under the different assayed conditions. Moreover, there also is a clear direct correlation between *S. kudriavzevii* proportions and glycerol production, another desirable enological characteristic of importance for wine quality because it contributes to wine body and astringency masking (Jolly et al., 2014). Glycerol and ethanol metabolism has been proven to differ in *S. kudriavzevii* with respect to *S. cerevisiae* (Arroyo-López et al., 2010; Pérez-Torrado et al., 2016). In fact, cryotolerant *Saccharomyces* species, such as, *S. kudriavzevii* and *S. uvarum*, have been proven to produce wines and ciders with higher glycerol contents than *S. cerevisiae* (Bertolini et al., 1996; Masneuf-Pomarède et al., 2010; Peris et al., 2016; González Flores et al., 2017), so their use could be of great interest for wine industry.

Among the strategies followed to favor *S. kudriavzevii* growth against *S. cerevisiae*, the co-inoculation with a proportion of *S. cerevisiae* lower than 10% and the sequential inoculation showed the more promising results. Air supply showed a synergistic effect in proportion *S. cerevisiae*/*S. kudriavzevii* 1:9, whereas it did not have a significant impact on the rest of the assayed inoculum proportions. These results agree with the fact that *S. cerevisiae* is better adapted to anaerobic conditions such as, wine fermentation, and air supply produces an imbalance in this environment, which promotes *S. kudriavzevii* survival. According to our results, it also seems feasible that a certain threshold in *S. cerevisiae* cell density is necessary to trigger *S. kudriavzevii* lack of viability. This also agrees with the previous observations indicating that the viability of a competitor strain is affected by its interaction with *S. cerevisiae* due to cell-to-cell contacts (Nissen et al., 2003; Arneborg et al., 2005; Branco et al., 2016; Pérez-Torrado et al., 2017b), or by microenvironment modifications produced by *S. cerevisiae* (Goddard, 2008). A rise in temperature due to the higher fermentative rate of *S. cerevisia*e (Goddard, 2008) can affect *S. kudriavzevii* viability (Arroyo-López et al., 2011).

In summary, the most promising results were obtained from the combination of different strategies for promoting *S. kudriavzevii* prevalence during wine fermentation, such as, co-inoculation with a low proportion of *S. cerevisiae* (<10%) or sequential inoculation together with limited aeration, resulting in an ethanol yield reduction as well as a higher glycerol production. Aeration requires costly additional technology, but it is already implemented in the wine industry (Vivas and Glories, 1996; Vidal and Aagaard, 2008) to improve wine quality by accelerating the transformations of phenols reducing the astringency.

Finally, these results have to be confirmed in real grape must to evaluate not only the effect of aeration on yeast physiology but also a potential effect on sensory profile. In addition, lower aeration rates can also be tested at industrial scale, particularly for *S. cerevisiae*/*S. kudriavzevii* proportions lower than 1:9. In addition a deeper understanding of the interactions among *Saccharomyces* yeasts, are also needed in order to finely tune the optimal use of these tools to reduce ethanol contents in wine.

## Author contributions

JA, GC, EB, and AQ conceived and designed the experiments. JA, AC, and GC performed the experiments. JA, EB, and AQ analyzed the data and wrote the paper.

### Conflict of interest statement

The authors declare that the research was conducted in the absence of any commercial or financial relationships that could be construed as a potential conflict of interest.
