# Supplementary material for: The Use of Mixed Populations of Saccharomyces cerevisiae and S. kudriavzevii to Reduce Ethanol Content in Wine: Limited Aeration, Inoculum Proportions, and Sequential Inoculation
Source: Front Microbiol. 2017 Oct 25;8:2087. doi: 10.3389/fmicb.2017.02087 (PMC5661026; doi:10.3389/fmicb.2017.02087)
Supplement: Supplementary file 1 [file Presentation1.pdf]

Supplementary Material

The Use of Mixed Populations of *Saccharomyces cerevisiae* and *S. kudriavzevii* to Reduce Ethanol Content in Wine: Limited Aeration, Inoculum Proportions and Sequential Inoculation

Javier Alonso-del-Real [1], Alba Contreras-Ruiz [1, 2], Gabriel L. Castiglioni [1,3†], Eladio Barrio[1, 2] and Amparo Querol [1*]

[1]Departamento de Biotecnología de los Alimentos, Grupo de Biología de Sistemas en Levaduras de Interés Biotecnológico, Instituto de Agroquímica y Tecnología de los Alimentos (IATA)-CSIC, Valencia, Spain**,** [2]Departament de Genètica, Universitat de València, Valencia, Spain, [3] Department of Food Engineering, School of Agronomy, Federal University of Goiás, Goiania, Brazil

*** Correspondence:** aquerol@iata.csic.es

1.1 Supplementary Figures

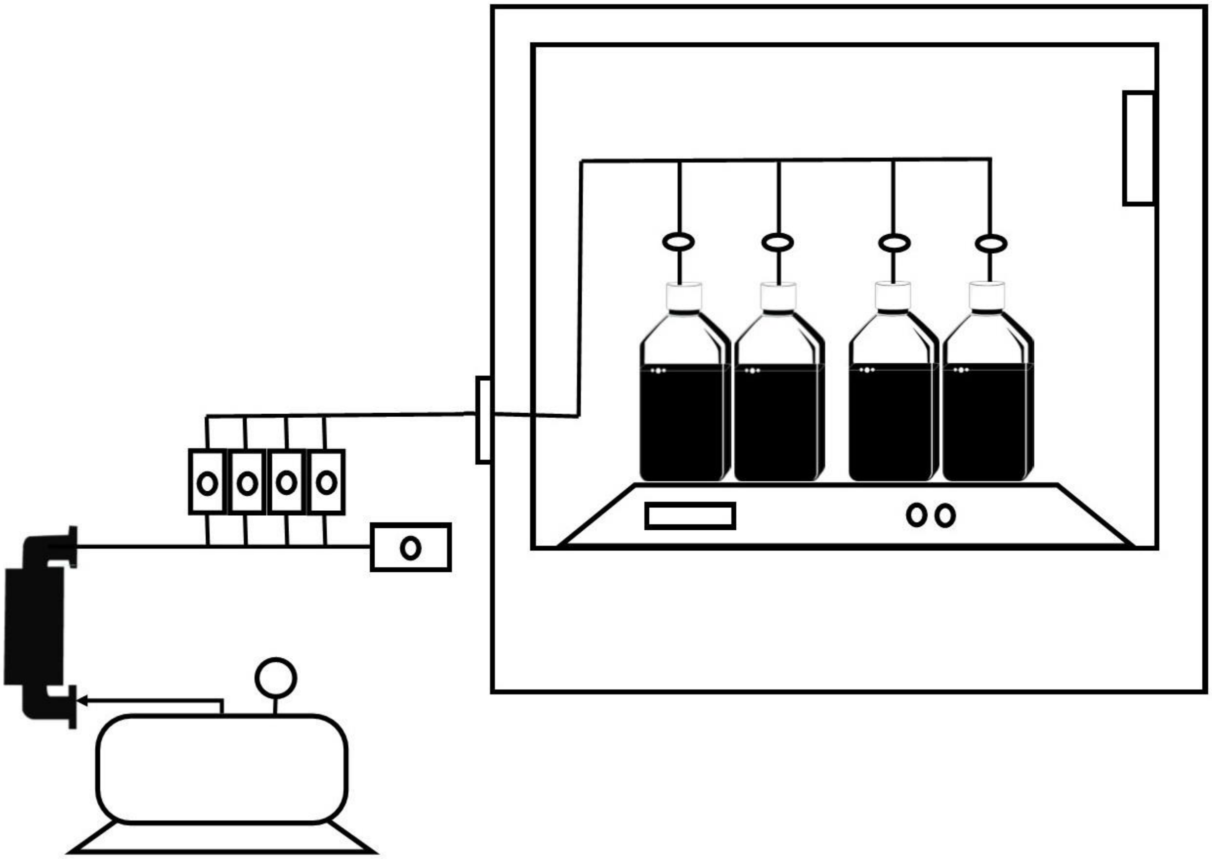

Supplementary Figure 1: Scheme of aeration system. (1) Compressed air generator; (2) Silicon tubes; (3) Filters; (4) Flow meter; (5) Set of flow regulators; (6) Shaker; (7) Incubator with temperature control; (8) Thermometer; (9) Bottle bioreactor.
